# Development of a Questionnaire to Measure the Attitudes of Laypeople, Physicians, and Psychotherapists Toward Telemedicine in Mental Health

**DOI:** 10.2196/mental.6802

**Published:** 2017-10-03

**Authors:** Peter Tonn, Silja Christin Reuter, Isabelle Kuchler, Britta Reinke, Lena Hinkelmann, Saskia Stöckigt, Hanna Siemoneit, Nina Schulze

**Affiliations:** ^1^ Institute of Applied Research in Neuropsychiatry Neuropsychiatric Center Hamburg-Altona Hamburg Germany

**Keywords:** screening, questionnaire, e-mental health, remote consultation, attitude to computers, physician expectations, telemedicine, online-intervention

## Abstract

**Background:**

In the field of psychiatry and psychotherapy, there are now a growing number of Web-based interventions, mobile phone apps, or treatments that are available via remote transmission screen worldwide. Many of these interventions have been shown to be effective in studies but still find little use in everyday therapeutic work. However, it is important that attitude and expectation toward this treatment are generally examined, because these factors have an important effect on the efficacy of the treatment. To measure the general attitude of the users and prescribers toward telemedicine, which may include, for instance, Web-based interventions or interventions through mobile phone apps, there are a small number of extensive tests. The results of studies based on small groups of patients have been published too, but there is no useful short screening tool to give an insight into the general population’s attitude. We have developed a screening instrument that examines such attitude through a few graded questions.

**Objective:**

This study aimed to explore the Attitude toward Telemedicine in Psychiatry and Psychotherapy (ATiPP) and to evaluate the results of general population and some subgroups.

**Methods:**

In a three-step process, the questionnaire, which is available in three versions (laypeople, physicians, and psychologists), was developed. Afterwards, it was evaluated by four groups: population-representative laypeople, outpatients in different faculties, physicians, and psychotherapists.

**Results:**

The results were evaluated from a total of 1554 questionnaires. The sample population included 1000 laypeople, 455 outpatients, 62 physicians, and 37 psychotherapists. The reliability of all three versions of the questionnaire seemed good, as indicated by the Cronbach alpha values of .849 (the laypeople group), .80 (the outpatients’ group), .827 (the physicians’ group), and .855 (the psychotherapists’ group).

**Conclusions:**

The ATiPP was found to be useful and reliable for measuring the attitudes toward the Web-based interventions in psychiatry and psychotherapy and should be used in different studies in this field in the future to evaluate and reflect the attitude of the participants.

## Introduction

There are many options currently available for telemedical contact or communication between the patients and the professionals. These include Web-based interventions, mobile phone apps, and remote screen calls. In psychiatry and psychotherapy, in addition to the conventional long-distance contact via phone calls or mail, the Web-based interventions (e-mental health) and mobile apps (m-mental health) are increasingly becoming important areas of care for the patients. Mental health, as a special part of the so-called telemedicine, covers two important areas. The first involves the information and education about the psychiatric disorders and psychosocial distress, and the second pertains to diagnostic and treatment tools. Many different Web-based services, in the form of email, social media, and other avenues, are accessible over the Internet almost worldwide. Also, a number of telemedical apps are available, especially via the Internet, for the somatic illnesses [1]. The everyday use (outside clinical trials) of Web-based tools for the diagnosis and treatment of mental illness, however, is not very popular yet [2,3]. For example, in the United Kingdom, there are only two computerized interventions recommended for the clinical treatment of psychiatric diseases (one against depression and another against panic and phobia) [4-6]. Phone calls or emails are regularly, though not frequently, used as the media of communication between the therapists and the patients in psychiatry or psychotherapy. Web-based interventions, however, are seldom used. Hence, it seems that there are barriers to the implementation of effective Web-based interventions that can help identify and overcome the issues related to mental health. The potential users recognize the rational advantages of the Web-based interventions such as anonymity, convenience regarding time, location, and the ability to do without a structured setting [7,8]. At the same time, the dropout rate among the users of computerized interventions of cognitive behavior therapy (CBT) is almost twice as high as compared with the dropout rate of face-to-face CBT [9]. The willingness to participate in a study on a *new* therapeutic concept seems to be good, but its application and use in the daily practice of therapy is very limited [10]. In a paper on the question of the meaningfulness of computerized interventions, only 22% of the participants indicated that the symptoms can be effectively improved [8]. Other studies show a negative attitude of the patients toward Web-based interventions, too. They have shown a low expectation of patients regarding whether their psychological complaints can be improved by computerized interventions, even to the conclusion that the patients do not want to use Web-based interventions in the future [11]. The current surveys on the attitudes toward and expectations from Web-based interventions are, in part, too comprehensive for a screening tool. Also, they do not reflect the attitude of the general population, focusing instead on selected populations (certain patient groups, Internet users with high affinity to the medium, etc). Another aspect is their outdated status, which renders them unable to represent the current possibilities that the Internet offers today.

Nevertheless, a considerable number of interventions via the Internet are actually already available, for example, to support the detection and treatment of posttraumatic stress disorder [4,12,13], anxiety disorders [14], or depression. In e-mental health, the currently available Web-based tools differ, among other things, regarding their depth of information—some only provide information about the disorder (psychoeducative), whereas others give different instructions for self-help (self-guided) or give some guidance from a psychotherapist about helpful activity (guided help) [1,15-17]. A few Web-based interventions allow additional contact with a psychotherapist via video call (Web-based counseling) or face-to-face care (blended) [18]. It is foreseeable that the Web-based interventions will play a growing role in all areas of medicine, even in psychiatry and psychotherapy, in the future [19]. However, the everyday use of the Web-based interventions does not correspond to the optimum use of the available resources. Also, the efficacy of the Web-based interventions does not lead to their use on a larger scale [10]. Therefore, it is necessary to find out the background of the probable users (both patients and the physicians or psychotherapists) to integrate this information into the development and distribution of new apps [20-27]. Furthermore, it also appears necessary, especially in the studies on efficacy and effectiveness, to examine the expectations of the users from such tools as a positive complement to the existing therapeutic spectrum. This is also important because, since the 1970s, it has been possible to show repeatedly that the patients’ expectations from and belief in the credibility of a therapeutic medium or process can significantly influence its effectiveness and efficacy [28]. Therefore, in the studies on the effectiveness of the Web-based interventions, especially when the participants are recruited from a website or other Web-based media, the sample may have been biased. This can be quantified at least by using a simple screening tool, which can show the data of the general population as comparative values.

To our knowledge, there is no short questionnaire that measures the concept of a favorable attitude toward telemedicine in psychiatry and psychotherapy, especially, but not exclusively, e-mental health. Only some difficult and complex measurements are published yet [7]. Therefore, we developed a questionnaire that can be used to evaluate the expectations and the attitudes of the users of such interventions, and we seek to publish the data of a large sample of the general population and some special groups (patients of different faculties, physicians, and psychotherapists) to present the comparative values.

## Methods

To develop the questionnaire, Attitudes toward Telemedicine in Psychiatry and Psychotherapy (ATiPP), we undertook a three-step process. We started by generating a set of items reflecting the attitude toward and the expectations from telemedicine in general and particularly, the use of telemedicine in mental health. We used the experience of a psychotherapeutic team and psychiatric consultants to find as many statements as possible, as well as depending on the available literature. For example, one statement was, “In general, telemedicine is a good addition to medical services.” The initial 15-item questionnaire was then used in a discussion process with the experts not otherwise involved in the development of the scale. The notes and comments that arose independently were included in the further development of the questionnaire. This was done in a modified Delphi process, which means that the experts discussed and developed the questions over several sessions [29]. In the modified Delphi technique, the panelists begin with a set of items to rate according to importance, rather than with an open-ended questionnaire. Here, these items were selected by the study team, drawing from various sources, including a literature review and interviews with content experts, patients, and the technical and medical specialists for Web-based interventions. The primary advantage of using this modification is that it typically improves the initial response rate [30]. The questions are posed in a series of rounds until a consensus is achieved or until it is obvious that the future rounds will not provide additional information. At each round after the first, the experts are provided with a feedback of anonymous comments from the panelists in the round before. The questions evaluated as substantive duplicates or evaluated as not relevant were excluded after the discussion when we developed the questionnaire. Then in a second step, we condensed the items to an 8-item questionnaire (from the initial 15-item questionnaire). A 5-point Likert scale was used with anchors ranging from 1=“I strongly agree” to 5=“I strongly disagree.” At this point, we developed three versions of the questionnaire so that the exact wording was matched to the individual function of the respondents. Consequently, the laypeople were asked what they prefer for themselves, whereas the practitioners were asked what they want to offer their patients.

We explain the questionnaire with the following sentences (ie, questionnaire for the laypeople):

The use of telemedicine as an online service, by phone call or, as a smartphone application, is lately being discussed more and more intensively. This is one of the consequences of improved technical opportunities on the one hand and scarcer resources on the other hand.

While radiology or dermatology are already using telemedicine for the purpose of image transmission for X-rays, CT scan, or skin photos, the use of telemedicine in the area of psychiatry and psychotherapy—despite some pilot projects worldwide—is still, largely, an uncharted territory.

In the context of a scientific study on psychiatric care research, we are investigating the attitudes of the users, the patients, and the referring physicians toward the telemedical services.

With the following questions, we would like to determine how you assess psychiatric or psychotherapeutic care via a telemedical offer, ie, via the Internet, email, telephone, or via a smartphone application.

We are also interested in whether you would use such an offer yourself.

Table 1 shows the 8 items of the three different versions of the questionnaire. The differences of the question texts are essentially explained by the functions of the respondents. Thus, the psychotherapists are specifically asked whether they consider telemedicine to be helpful in somatic medicine because the Delphi process in developing the questionnaire showed that the psychotherapists did not reflect the somatic applications of telemedicine in the vast majority. Also in Question 6, the differences arise from the fact that the psychotherapists are in contact with the patients who are or have already been in a face-to-face therapy, that is, only those who have already been treated or who are waiting for treatment are affected, whereas in the case of the physicians, the patients were treated with many diagnoses but usually not in a psychotherapeutic setting there. In Question 3, we asked the patients for a successful treatment in colloquial speech, while we asked the same from the physicians and psychotherapists in professional wording.

The resulting questionnaire was then used in a study with four groups of participants—the physicians, the psychotherapists, the outpatients in the waiting areas of various medical disciplines, and a representative population sample in a telephone interview.

The population sample was based on the telephone number ranges provided by the German Federal Network Agency. This number range includes all telephone numbers in the Federal Republic of Germany, including mobile telephone numbers. We used the nationwide sample of telephone numbers. A nationwide list of the German Market Research Association (ADM) was used for the telephone survey. The connection data are presorted here according to various aspects (eg, regions) and can be appropriately used in a representative sample. As the share of households that do not publish their telephone numbers is increasing steadily, the ADM telephone sample contains both registered and generated numbers. The generation of numbers was done with a process similar to Brick [31]. In this study, we collected 50.00% (500/1000) of the general population with the generated numbers and 50.00% (500/1000) with the published numbers. This procedure ensured that both the regional distribution of the calls as well as the allocation to age groups, etc, corresponded to the population distribution in Germany; thus, a representative study sample could be collected.

The group of patients was selected from four outpatient clinics—two general practitioners’ practices, a gynecology practice, and a center for neurology. The physicians and the psychotherapists were selected through a Germany-wide mailing action. We randomized 200 physicians from different disciplines and 200 psychotherapists from the nationwide database of the physicians and therapists by the means of a computer-generated randomization. We distributed the questionnaire as a printed version. The return was free and anonymous, and no reward was given.

We analyzed the data from the measurement by testing the reliability. We had expected the items to be presented in a graduated form as one dimension—acceptance or attitude. In particular, we assumed that the questions are in a relationship with each other, and we examined whether the individual questions are replaceable or dispensable.

**Table 1:** The 8 items of the Attitude toward Telemedicine in Psychiatry and Psychotherapy (ATiPP) questionnaire.

Number	Laypeople	Physicians	Psychotherapists
1	Generally, telemedicine is a good addition to the medical services.	Generally, telemedicine is a good addition to the medical services.	Telemedicine in somatic medicine is a good addition to the medical services.
2	For psychiatric or psychotherapeutic issues or mental illness, patient information via Internet or telemedicine is very helpful.	For psychiatric or psychotherapeutic issues or mental illness, patient information via Internet or telemedicine is very helpful.	For psychiatric or psychotherapeutic issues or mental illness, patient information via Internet or telemedicine is very helpful.
3	A successful treatment of the patients with mental illness via Internet or telemedicine is possible.	An effective treatment of the patients with mental illness via Internet or telemedicine is possible.	An effective treatment of the patients with mental illness via Internet or telemedicine is possible.
4	The bridging of the waiting time for an appointment with a psychiatrist/psychotherapist by using the Internet services or telemedicine is a sensible option.	The bridging of the waiting time for an appointment in psychiatry/psychotherapy by using the Internet services or telemedicine is a sensible option.	The bridging of the waiting time for an appointment in psychiatry/psychotherapy by using the Internet services or telemedicine is a sensible option.
5	Aftercare and counseling after a presence therapy by a psychiatrist or psychotherapist through contact via the Internet or email or telephone are realizable.	Aftercare and stabilization after a presence therapy by a psychiatrist or psychotherapist through contact via the Internet or or email or telephone are realizable.	Aftercare and stabilization after a presence therapy by a psychiatrist or psychotherapist through contact via the Internet or email or telephone are realizable.
6	I would make use of Web-based interventions or telemedicine without an accompanying face-to-face therapy in the case of a mental illness.	I would absolutely recommend my patients with psychiatric or psychotherapeutic treatment needs a Web-based intervention or telemedical support, if such were to be offered for the clinical picture.	For my own patients, I would offer support and intervention via the Internet or telephone.
7	An online therapy via the Internet services or telemedicine is only sensible as an addition to face-to-face therapy.	In addition to a face-to-face therapy, an accompanying psychoeducational or psychosocial or additional intervention via the Internet is sensible.	In addition to a face-to-face therapy, an accompanying psychoeducational or psychosocial or additional intervention via the Internet is sensible.
8	An online therapy through the Internet services or telemedicine can only work effectively with live contact with a therapist through video calling and email or chat.	An online therapy through the Internet services or telemedicine for mental illness can only work effectively with live contact with a therapist through video calling and email or chat.	An online therapy through the Internet services or telemedicine for mental illness can only work effectively with live contacts to a therapist through video calling and email or chat.

All statistical data were analyzed with the open source program R, version 2.2.4 (R-Core-Team).

The chair of the Ethical Commission of the Board of Physicians (Institutional Review Board) in Hamburg, Germany, did not consider ethical approval to be necessary because of the anonymous type of survey of the participants.

## Results

The participants of the first survey answering the questionnaire as a population-representative random sample were 51.40% (514/1000) female. The age was distributed according to the proportion in the population and divided into 10-year clusters. The smallest age group with 12.60% ranged from 16 to 25 years, followed by 14.30% (143/1000) from 26 to 35 years, 15.00% (150/1000) from 36 to 45 years, 17.70% (177/1000) from 46 to 55 years, 16.00% (160/1000) from 56 to 65 years, and 24.40% (244/1000) for the age group of 65 years and older.

The Cronbach alpha value for the 8 items reached the conventional standards for scale reliability (alpha=.849), and no item reduction seemed meaningful. We used the questionnaire version for the laypeople in this particular study.

In the next part of the study, we included a sample of outpatients in the waiting rooms of different physicians—the general practitioners, the gynecologists, and the neurologists. Here, 324 participants (of 624 asked) completed the questionnaire; 65.7% (213/324) of them were female. The age showed a distribution of 13.7% (48/324) in the age group of 16 to 25 years, 26.9% (94/324) for 26 to 35 years, 17.4% (61/324) for 36 to 45 years, 17.4% (61/324) for 46 to 55 years, 11.1% (39/324) for 56 to 65 years, and 13.4% (47/324) for those aged 65 years and older.

The Cronbach alpha value for the 8 items reached the conventional standards for scale reliability (alpha=.80), and no item reduction seemed meaningful. We also used the questionnaire version for laypeople. Table 2 shows the results of the study with the mean (M) and standard deviations (SD). Due to the low sample size of the psychotherapists, we added the physicians and the psychotherapists here to the category of professionals.

**Table 2:** The mean and the SD of the items and the overall measures. The questions are coded from “1=strongly agree” to “5=strongly disagree,” and so, the lower values are in better agreement with the question whereas the higher values indicate more disagreement. The total scale is the mean of the 8 single-item values.

Item	General population, mean (SD^a^)	Professionals, mean (SD)
1	3.06 (1.27)	2.26 (1.09)
2	3.04 (1.23)	2.54 (1.14)
3	3.59 (1.26)	3.45 (1.20)
4	2.94 (1.29)	2.64 (1.26)
5	2.95 (1.28)	2.89 (1.14)
6	3.97 (1.28)	2.89 (1.33)
7	2.84 (1.37)	2.39 (1.13)
8	2.96 (1.26)	2.67 (1.24)
Total scale	3.17 (0.89)	2.64 (0.88)

^a^SD: standard deviation.

**Table 3:** The scale reliability with single-item deficient (Cronbach alpha deleted for one item) and selectivity coefficient (corrected part-whole-correlation).

No.	Laypeople		Physicians		Psychotherapists	
	Cronbach alpha	Selectivity	Cronbach alpha	Selectivity	Cronbach alpha	Selectivity
1	.829	.603	.887	.663	.842	.565
2	.826	.627	.883	.716	.814	.791
3	.837	.532	.876	.784	.828	.687
4	*.816*	*.706*	.879	.752	.829	.669
5	.817	.701	.879	.755	.825	.729
6	.843	.484	.873	.811	.818	.745
7	.838	.533	*.886*	*.683*	.833	.644
8	.840	.511	.917	.325	.897	.083

The third part of our study was directed toward the physicians. The group size was 92, with 47% (43/92) of the physicians being female (and the distribution of age showing 1% for those under 36 years (1/92), 11% (10/92) for those in the age range of 36 to 45 years, 43% (39/92) for 46 to 55 years, and 44% (41/92) for those aged above 55 years. We used the questionnaire version for the physicians in this part of the study. The Cronbach alpha value was .827, and so, no correction of the questionnaire was necessary at this point.

In the last part of the study, we surveyed the psychotherapists. This group consisted of 36 participants, with 67% female and the distribution of age revealing 5% (2/36) to be less than 36 years, 5% between 36 and 45 years (2/36), 36% (13/36) in the 46 to 55 years age range, and 53% (19/36) being older than 55 years. Reliability was good; we found a Cronbach alpha value of .855. Here, we used the questionnaire version for the psychotherapists.

The comparison of the 3 versions of the questionnaire shows no relevant differences; the reliability is nearly similar. Only in the questionnaire for the psychotherapists, item 8 (*Therapy through the Internet services/telemedicine for mental illness can only work effectively with live contacts with a therapist through video calling and email/chat.*) shows a selectivity under 0.1 and must, therefore, be questioned.

The analysis of the Cronbach alpha values by deleting the singular items and selectivity are shown in Table 3.

Question 4 shows the clearest significance (*P*=0.001) among the laity in the positive assessment; also in the Cronbach alpha a good consistency is confirmed here.

A presentation and comparison of the results of all aspects of the study will be done and published.

## Discussion

We developed an 8-item questionnaire with three versions that cover the important aspects of the attitudes and expectations of the laypeople, the physicians, and the psychotherapists toward the telemedical interventions in psychiatry and psychotherapy (eg, e-mental health, Web-based interventions, and phone interventions). This is the first time that a really short and clear questionnaire has been constructed to look at the attitudes toward Web-based interventions and telemedicine in mental health. This was important because of the influence that the patients’ expectations and attitudes toward a diagnostic and therapeutic process or tool have on the efficacy and the results of these processes or tools.

Earlier studies have shown that a variety of telemedical interventions, especially Web-based interventions, are available in many countries. And it seems that the number, especially of Web-based interventions or mobile phone apps, is growing daily. However, despite the high demand for psychological support, these resources are only used to a limited extent in the everyday therapeutic work. It is unclear whether this is related to the attitude toward Web-based interventions and other telemedical services. However, previous work has already shown, for example, with the help of the credibility and expectancy questionnaire, that therapeutic success is closely related to the attitude toward therapy. So far, such investigations with regard to the Internet-based interventions have been carried out with some limitations. Schröder et al [32] had examined the patients and the psychotherapists in the context of telemedical care, but there has not been any recruitment of a sample of the general population. Gun et al [33] have also interrogated the disorder-specific groups, even in a large sample. Musiat et al [7] and Wangberg et al [34] have attempted to investigate a non-disorder-specific group, but as a population is only accessible by social media, those people questioned can be estimated as being open-minded toward the Internet. There is no doubt that a similar bias is to be feared in the evaluation of the therapeutic approaches on the Internet—especially if those who are positively opposed to this medium will take part in these studies. To achieve a broad effect and to use the Web-based interventions not only as niche products, the patients who are not “early adopters” must also be reached. Also, the (particularly) positive expectation of the participants in the studies—which may differ significantly from the general population—must be considered. Checking this possible bias will be helpful for examining the attitudes of the participants and the patients in a study and for a comparison with the general population. This can be done with a questionnaire such as the ATiPP—because it is short and quick, and there are growing numbers of different participants and groups we had checked (and will publish the data in the near future).

Actually, we had a sufficient number of participants among the general population and the patients, but the physicians and the psychotherapists were also included in the first evaluation. The comparison of the three versions indicates that the same contents are measured, reflecting the positions of the participants. The collection and analysis of more data from the participants using the ATiPP is currently underway; we are testing the results in other groups and with other subjects. In addition, the questionnaires published so far, such as Credibility/Expectancy Questionnaire (CEQ) [28], will be checked against the ATiPP in smaller groups. In principle, we assume that the attitudes toward the use of Web-based interventions and other telemedical services can be adequately examined and recorded with the ATiPP.

However, we are conducting a study to create pre- and postdata using the questionnaire in a sample of laypeople and professionals before and after participating in a three-lesson e-learning session with a focus on e-mental health interventions. We expect a further confirmation that the questionnaire may also reflect the developments in the attitudes toward and expectations regarding e-mental health. There are currently some limitations. Thus, there is a lack of a factor analysis, which we have prepared but have not yet concluded with regard to the evaluation of the subgroups. It is, thus, not clear whether the questionnaire actually measures a dimension, as the reliability values suggest, or whether the two dimensions (credibility and expectancy) are also collectively taken as an attitude in ATiPP, which the CEQ as somewhat a comparable instrument might at least suggest.

In conclusion, we recommend the use of the questionnaire to measure the attitude toward and expectations regarding e-mental health. The growing number of Web-based interventions needs a growing knowledge of the potential users—the laypeople as well as the physicians and the psychotherapists. They must be informed about the bugs and hints as also the positive effects in order for them to accept the Web-based interventions and to understand the strengths and limitations of e-mental health. Only if the attitudes toward the Web-based interventions become more positive and the expectations of the users correspond to the actual good results of the interventions, a therapeutic use of telemedicine, especially e-mental health and m-mental health, will occupy a wider space in everyday work and life. This could be achieved through training programs, and the effect of such programs can be evaluated by the use of our questionnaire.

## Abbreviations

ATiPP: Attitude toward Telemedicine in Psychiatry and Psychotherapy
CBT: cognitive behavior therapy
CEQ: Credibility/Expectancy Questionnaire
SD: standard deviation
